# A theoretical exploration of polyvagal theory in creative arts and psychomotor therapies for emotion regulation in stress and trauma

**DOI:** 10.3389/fpsyg.2024.1382007

**Published:** 2024-05-22

**Authors:** Suzanne Haeyen

**Affiliations:** ^1^GGNet, Centre for Mental Health, Scelta, Centre of Expertise for Personality Disorders Apeldoorn, Warnsveld, Netherlands; ^2^Research Group for Arts and Psychomotor Therapies in Health Care, Academy of Health & Vitality, HAN University of Applied Sciences, Nijmegen, Netherlands; ^3^KenVaK, Research Centre for Arts Therapies, Heerlen, Netherlands

**Keywords:** polyvagal theory, creative arts therapies, psychomotor therapy, emotion regulation, stress, trauma

## Abstract

Polyvagal theory advocates for working with the body, becoming aware of the body and connecting with the senses. Similarly, paying attention to and influencing one’s physical and sensory experience is a core aspect of the creative arts and psychomotor therapies. Polyvagal theory offers opportunities for strengthening resilience by treating emotion-regulation problems, stress, and trauma, as well as restoring regulation of the autonomic nervous system. Paying attention to and influencing physical and sensory experiences are core aspects of creative arts and psychomotor therapies. This theoretical paper explores how polyvagal theory can serve as a foundational theory and support the creative arts and psychomotor therapies for emotion regulation in stress and trauma. A number of pillars in polyvagal theory have links with arts therapies, such as an emphasis on physical and sensory experience in situations of safety or threat. This theory may offer insight into the role of the body in stressful situations, the role of co-and self-regulation, and thus the functioning of and the rationale for use of creative arts and psychomotor therapies. Through interventions focused on promoting healthy autonomic responses and regulating physiological responses, clients can learn to better regulate and process their emotional experiences. Although this could be broadly useful, it would seem particularly promising in therapies focused on stress and trauma. This article provides an introduction to polyvagal theory and outlines how it can serve as an explanatory, hypothetical model for the working mechanisms that underlie creative arts and psychomotor therapies. The application of PVT in creative arts and psychomotor therapies will be explored by describing techniques for “noticing and naming” and “learning to change,” as well as by highlighting the role of PVT in the therapeutic relationship. It provides case examples and discusses the role of creative arts and psychomotor therapies for stress regulation and resilience conceptualized in line with the polyvagal theory.

## Introduction

In society and in many kinds of therapy, the rational mind and cognition are considered pre-eminent, while the body and emotions are neglected. This division between the brain/mind and the nervous system is not new. According to the 17th century philosopher Descartes, the mind provides enlightenment; it helps us to think clearly, which in turns enables us to progress in our lives. Descartes argued that mind and body are distinct; two separate entities, whereby the physical was seen as less valuable. The body was a source of temptations that one could best ignore. This type of thinking continues to influence how brain and body are viewed today: reason is often prioritized over physicality. Polyvagal theory (PVT) approaches brain and body not as “either/or” but as interconnected systems (Porges, 1995, 2019, 2021, 2023).

PVT advocates for working with the body, becoming aware of the body and connecting with the senses. Paying attention to and influencing physical and sensory experience is, likewise, a core aspect of creative arts and psychomotor therapies (e.g., Malchiodi, 2012; Haeyen, 2018; Malchiodi, 2020). PVT offers opportunities for treating emotion-regulation problems, stress, and trauma. The focus is on restoring regulation of the autonomic nervous system, thus offering tools for strengthening resilience. This article considers whether and how PVT can underpin the creative arts and psychomotor therapies and be applied in treatment focused on stress and trauma. Although some physiological tenets of PVT are subject to dispute (Steffen et al., 2022; Grossman, 2023; Verkuil, 2023), the experiences of clients and therapists in clinical practice suggest that it may provide tools that support personal recovery.

In this theoretical paper PVT will be introduced by highlighting important themes like the hierarchy in the autonomic nervous system (ANS), neuroception and stress regulation in therapy. Next, PVT will be explored as a hypothetical foundation for creative arts and psychomotor therapies. The clinical application of PVT in creative arts and psychomotor therapies will be described in case examples and explored by describing specific techniques for “noticing and naming,” techniques for “learning to change” and its role in the therapeutic relationship (Haeyen, 2023). The paper aims to focus on gaining insight into the role of the body in stressful situations, the role of co-and self-regulation, and thus the functioning of and the rationale for use of creative arts and psychomotor therapies. The paper concludes with a discussion of the use of PVT in creative arts and psychomotor therapies for emotion regulation in stress and trauma.

## Polyvagal theory

**Figure 1:**
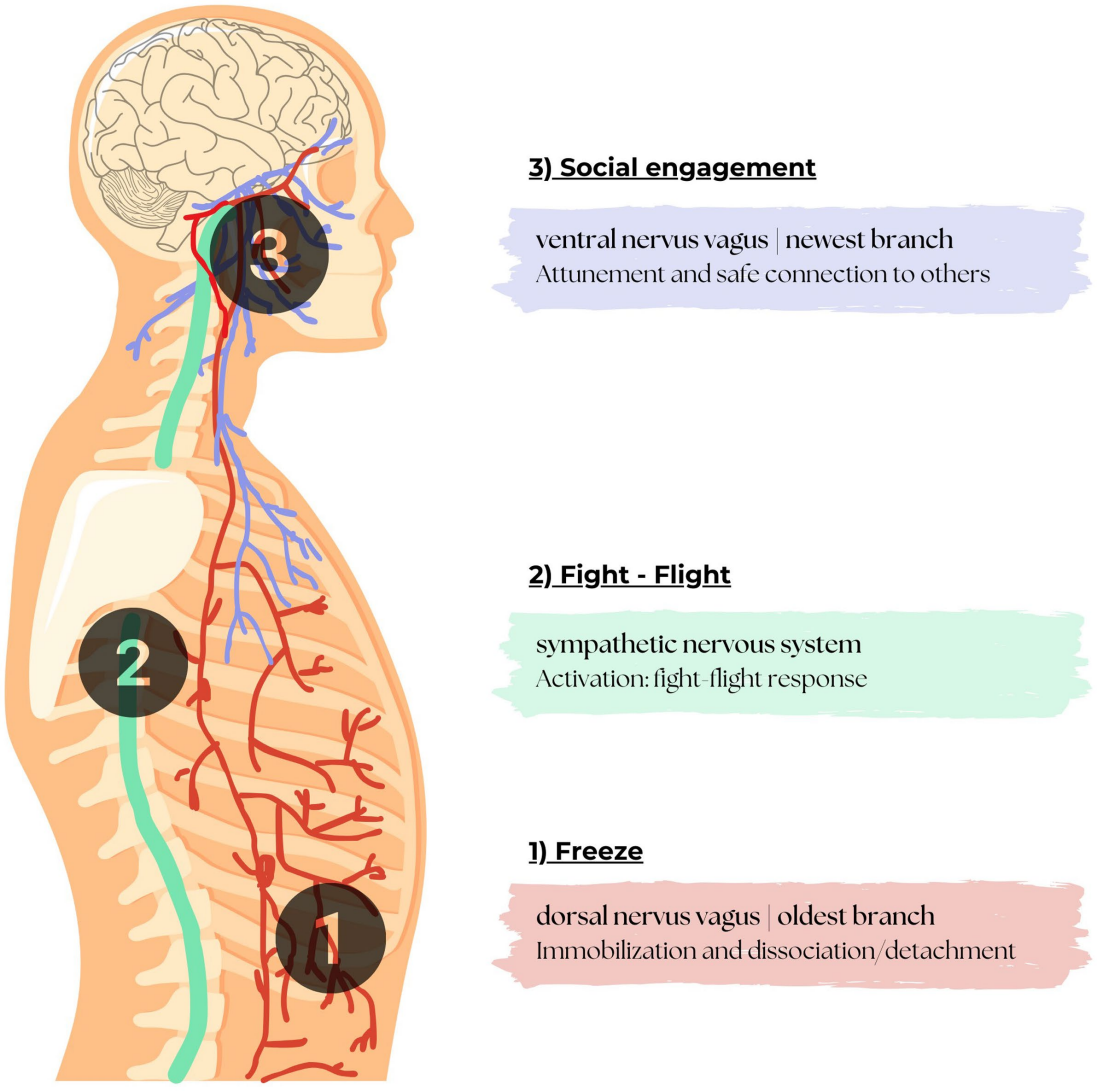
Dorsal (1), sympathetic (2) and ventral vagal nerve branch (3).

Polyvagal theory (PVT) was proposed by Porges (1995), who pointed out that approximately 80% of nerve signals are sent from the body to the brain, and only 20% from the brain to the body. This means that bodily signals-sensations, emotions, and physiological changes-provide much of the information the brain needs to function. These signals are not only informative, but also regulatory. PVT conceptualizes how this two-way communication between body and brain works (Figure 1). The phylogenetically ordered response hierarchy regulates autonomic state adaptation to safe, dangerous, and life-threatening environments (Porges, 2023).

*Polyvagal* refers to multiple vagal pathways (*poly* = many, *vagal* = involving the vagus nerve). PVT describes a connection between the body’s (autonomic) responses and the brain, based on the evolution of the ANS. It describes the role of the ANS in social behavior, emotions, and stress responses, focusing on safety or threat.

The ANS is regulated by the hypothalamus and prepares the body for action and recovery after action. The vagus nerve (the “wandering nerve”) is a component of the parasympathetic NS, containing 80% afferent sensory neurons and 20% efferent motor neurons (Engelen et al., 2023). The vagus is a cranial nerve that exits the brainstem and travels to several organs within the human body. It is the primary neural pathway of the parasympathetic nervous system. The vagus nerve influences heart rate and breathing, thus playing an important role in the regulation of emotions, stress, and social behavior (Porges, 2021). The ANS, consisting of a sympathetic and a parasympathetic component, regulates many bodily functions without conscious control. PVT adds a third component, the social engagement system, which is important for human interaction. The vagus is primarily a sensory nerve. The autonomic state functions as an intervening variable (Porges, 2023).

### Hierarchy in the nervous system

According to PVT (Porges, 1995, 2021), the ANS evolved in the context of security and threat. The Polyvagal Theory (PVT) initially described how evolution shapes the nervous system to support social connections and cooperation among vertebrates. This theory conceptualizes changes in the autonomic nervous system (ANS) across species, highlighting its role in behaviors like mother-infant bonding. By understanding how the ANS influences social behavior, the theory aims to bridge gaps in scientific understanding, offering a unified perspective on the mind-brain–body connection and its role in promoting either social bonding or defensive responses. Basically, hypotheses driven by PVT are related to the documentation that the mammalian ANS has a built-in hierarchy of autonomic reactivity based on phylogeny that is mirrored in embryological development. Depending on the state of the ventral vagus, autonomic regulation may either function hierarchically or antagonistically (Porges, 2023).

Given the complexity of the theory, the basics of PVT have not always been accurately transmitted by practitioners representing applied areas (e.g., medicine, education, business, and psychotherapy) who have become interested in the theory but who are not educated in the foundational sciences upon which PVT is dependent. There is also some criticism, sometimes based on misunderstandings of the scientific foundation upon which the theory is based (Neuhuber and Berthoud, 2022; Taylor et al., 2022; Porges, 2023).

### Stress regulation: safety first

Safety is one of the basic human needs. The perception of threat evokes an immediate and automatic response in the ANS. According to PVT, the search for safety is closely linked to the functioning of the ANS and three neural circuits that form a phylogenetically ordered response hierarchy that regulate autonomic state adaptation to safe, dangerous, and life-threatening environments. Based on its neuroscience, the vagus nerve is the tenth (X) cranial nerve which has a ventral and dorsal pathway that are involved with parasympathetic nervous system control of the heart, lungs, and digestive tract. PVT describes the structure of the nervous system according to three distinct nerve branches: an old branch, a more recent branch, and the newest branch (Porges, 1995, 2021; Dana, 2020). These different nervous systems of the ANS contribute to evoking active (i.e., fight or flight) versus passive (i.e., tonic immobility, emotional shutdown) defensive responses (Terpou et al., 2019).

The oldest branch, the *dorsal vagal branch*, runs from the brain stem at the back of the body down the front of the spine, where it connects to a number of organs. This nerve branch provides for immobilization, i.e., the shutting down of functions or “freezing,” in the event of danger (Porges, 1995, 2021; Dana, 2020). When this system is active, it manifests itself in immobilization and dissociation. Emotional shutdown is thought to involve the complete detachment from one’s sense of self from their body state (Feeny et al., 2000; Schauer and Elbert, 2010; Kozlowska et al., 2015).

The sympathetic system runs through the spine to a number of organs. This system is regulated by activation of the sympathetic nervous system. This is a neurotransmitter and hormone-driven system. When a stressful situation arises, the sympathetic nervous system allows us to take action: fight or flight. The many connections between this branch and multiple organs explain why stress can manifest itself in physical symptoms.

The newest branch, the *ventral vagal branch*, runs from the brain to the front of the body, from face to diaphragm. This ventral nervus vagus activates the ability for one to attune to and safely connect with others on a social level.

*Illustration created for this paper with permission to use based on* Adult Medical Specialists (AMS) (2023). When people feel safe, the different parts of the ANS work together to promote a state of relaxation, security, and connection (Porges, 1995, 2021; Dana, 2020). When there are signals of stress or danger, people generally respond first from the newest branch: connect with the other and set boundaries. If this does not help, the sympathetic system triggers a fight or flight response. If this fails too, the oldest system activates, leading to immobilization (blacking out, fainting, dissociation, or even paralysis). The physical states identified by Porges (2019) based on cooperation between the different nerve branches encompass social engagement/connection; active avoidance behavior; fight mode; freeze/passive avoidance behavior; shutdown; flow/pleasure/energy; and intimacy. The active versus passive responses are characterized by the increased and the decreased expression of the sympathetic nervous system. Moreover, active and passive defenses are accompanied by primarily endocannabinoid and opioid-mediated analgesics, respectively (Terpou et al., 2019). The periaqueduct grey is a necessary component of the freeze response (Roelofs, 2017). Also, pain-processing circuitry refers to the means by which the body signals modulate pain in alignment with the demands of the current fear provoking situation. Changes to these systems evoke dissociable defensive states. This is also described as the defense cascade model (Terpou et al., 2019). The defense cascade model aims to better understand the pathological presentation of defensive responses in PTSD with a focus on the functioning of lower-level midbrain and extended brainstem systems. Physiological adaptations are important in the symptom presentation of PTSD and its dissociative subtype.

### Neuroception

The ANS is constantly scanning for safety: in our environment, our relationships, and ourselves. This happens rapidly and mostly unconsciously. As a result, many stimuli do not enter our consciousness, but do influence our reactions. Porges (2021) calls this process of responding to subliminal signals *neuroception*. Neuroception is the reflexive detection of risk which triggers an adaptive autonomic state to optimize survival (Porges, 2023). It determines which part of our ANS is active. It involves internal signals (*interoception*: body temperature, heart rate, energy level, etc.) and external signals (*exteroception*: sensations, perceptions of the other person or the environment, etc.). The central nervous system (CNS) has a role in integrating interoceptive and exteroceptive information. The central nervous system’s responsibilities include receiving, processing, and responding to sensory information. Neuroception affects a person’s *perception*, which refers to phenomena that a person consciously perceives and imbues with meaning. Every individual views the world through the lens of their own experiences and life history, and creates their own narrative based on those cues. Neuroception determines how our ANS responds.

## Stress regulation in therapy

While some stress is important for healthy development, chronic stress has pathological consequences. There are long-term negative physiological effects of stress, such as heart and respiration rates, on the brain and neuroendocrine systems (Dunlavey, 2018). PVT includes the response systems that interact with the autonomic nervous system (i.e., neuropeptides, HPA axis and the immune system) to mediate physiological state in order to promote or limit social behavior (Porges, 2001). The social engagement system is intimately related to stress reactivity. In addition, the anatomical structures involved in the social engagement system have neurophysiological interactions with the hypothalamic–pituitary–adrenal (HPA) axis. The neuropeptides of oxytocin and vasopressin, and the immune system (Porges, 2009, 2023). The physiological state is an intervening variable that may buffer or exacerbate the effective impact of stress and trauma. Physiological adaptations and autonomic dysregulation are central to the PTSD diagnosis. PVT places the focus for clinical treatment on the physiological state. Successful therapy aims to change a patient’s perception of their physiological state, the neuroception, from a bias of reactionary and defensive in nature to positive and prosocial (Porges, 1995). Through the promotion of bodily attunement via neuroception, an individual may be able to identify somatic markers that denote a transition from a prosocial to a defensive state following threat or trauma-related processing. In turn, the patient may learn to exert cognitive control to attenuate the physiological adjustments which signal a defensive state (Terpou et al., 2019). There are three levels of stress response, also called the “stress ladder” (Porges, 1995, 2021; Dana, 2020):

1. Safety and social connection (ventral vagal state): the heart rate is calm and regular
2. Mobilization (sympathetic state): the heart rate accelerates and the muscles tighten in preparation for “fight or flight”
3. Immobilization (dorsal vagal state): the heart rate slows and the body “freezes”

**Figure 2:**
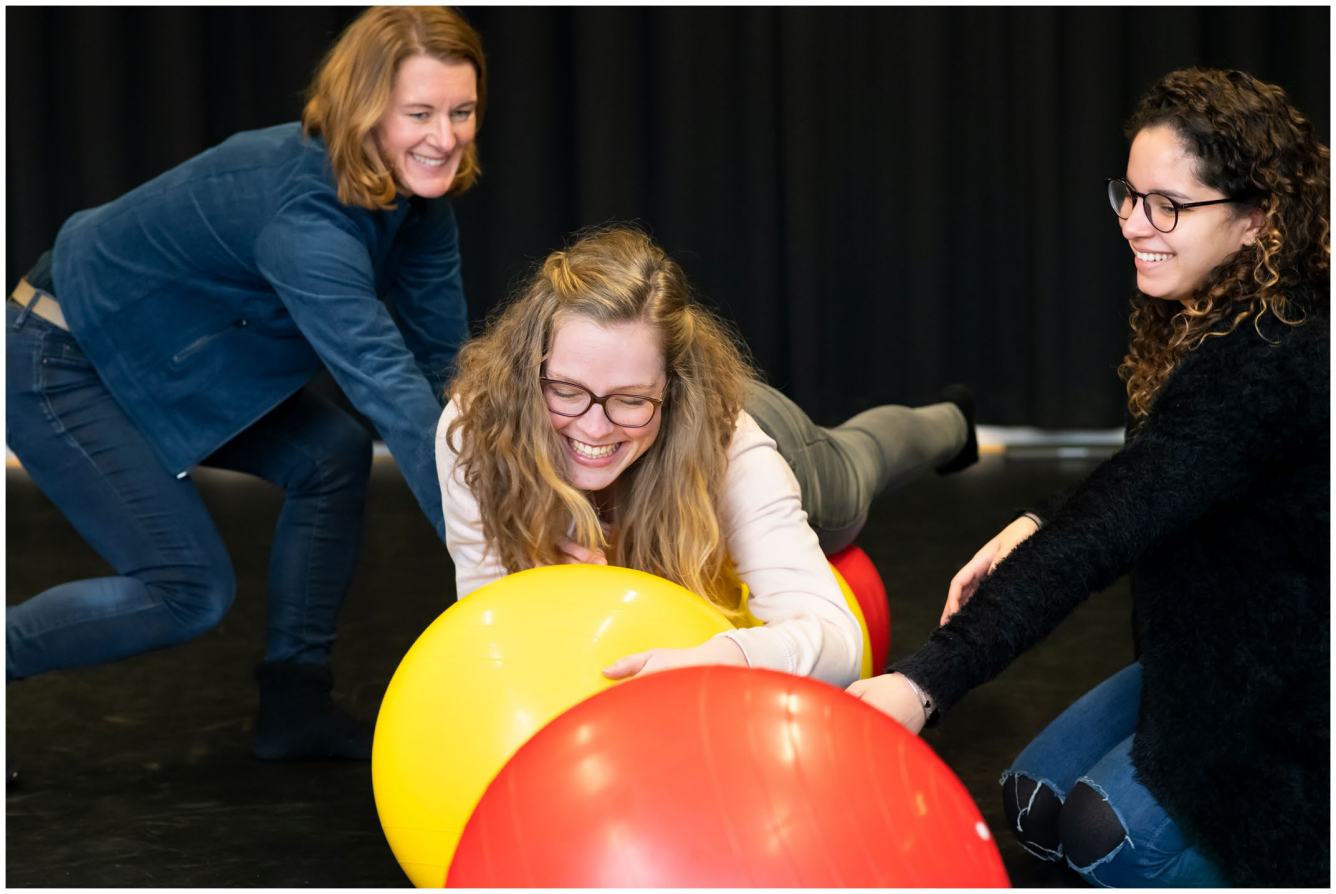
Art therapists in training experiencing safety and social connection during play.

**Figure 3:**
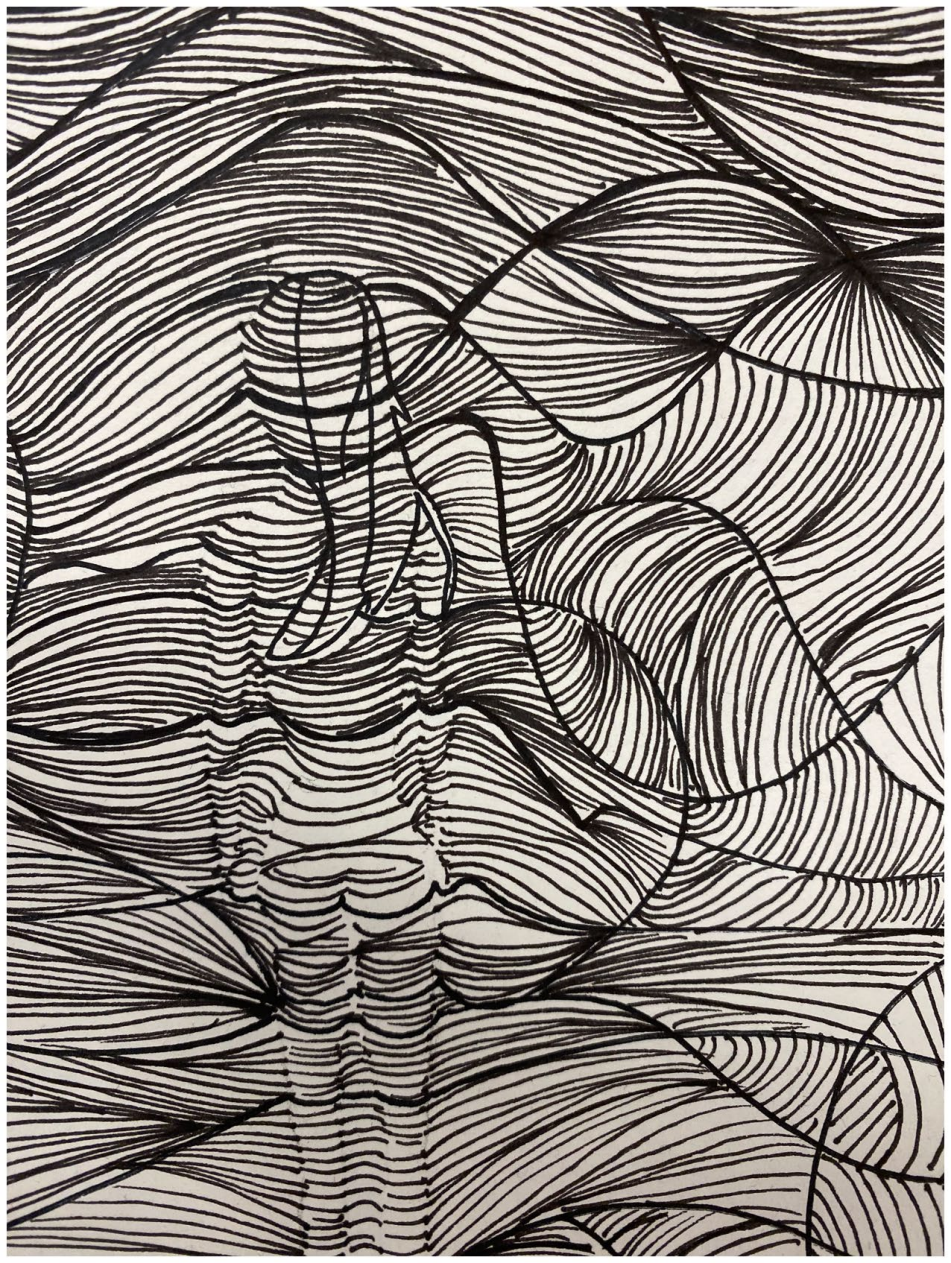
Immobilization symbolically imagined in art therapy: “I am not here” (Norah, age 27).

Different levels of stress responses can be combined, like the combination of the ventral and sympathetic vagal state in social play (see Figure 2). A stress response can be present in action or symbolically represented in art work, as is shown in Figure 3.

Therapy can strengthen an individual’s ability to switch between these three levels, which is essential for healthy regulation of emotions and behavior. In cases of stress and trauma, this ability is often disturbed: the person is unable to “switch gears” or calm down, under-or overreacts, feels either flat or hyper-alert, or is always in fight or flight mode. Dissociative or depersonalized states may be active when a client is unable to do anything, is making very flat art work, or is present in an absent way. Sometimes, doodling or presenting ‘absence’ in an image (see Figure 3) can also be the case. Emotional shutdown and detachment from one’s sense of self from their body state can take different forms. Switching between the stress-response levels requires self-regulation, co-regulation, and flexibility. In PVT, the term “vagal brake” refers to the inhibitory influence of the vagus nerve on the sympathetic nervous system (SNS) activity, being responsible for promoting relaxation and restoration. When the vagal brake is engaged, it slows down or inhibits the fight-or-flight response of the sympathetic nervous system, allowing the body to relax and engage in social engagement behaviors. This activation of the vagal brake is associated with feelings of safety, social connection, and physiological calmness. The efficiency of the vagal brake is related to clinical symptoms (Dale et al., 2022).

*Self-regulation* refers to an individual’s ability to calm down and feel safe. *Co-regulation* is the interaction between individuals that contributes to stress regulation. When individuals feel safe and connected in one another’s presence, the vagus nerve is activated, promoting relaxation and recovery. A child who cannot yet self-regulate first learns co-regulation with the attachment figure through comfort/soothing, body contact, etc. Self-regulation and co-regulation are both important to be able to regulate physiological and emotional responses, thus creating safety and connection.

Trauma may impact the ANS because co-regulation was unavailable, unpredictable, or unsafe, and self-regulation inadequate or unsuccessful (Dana, 2020). In that case, adaptive survival responses override social engagement and self-protection patterns. Porges (2021) states that trauma is a chronic disruption of connectedness. In trauma, neuroception is highly active for signs of danger. PVT offers opportunities for treating trauma, focusing on restoring regulation of the ANS. To this end, it is important to draw attention to signals: “What do you notice in your body, what emotions do you feel, and what makes you think/feel that way right now?.” Attention to bodily signals helps to find a way to emotionally connect in case of emotional shutdown and detachment from one’s sense of self. This process may be difficult and can take a long time in case of (structural) dissociation. Healing from trauma is indicated by one’s ability to be in an optimal state of arousal while experiencing affective sensations/emotions in the body (Kearney and Lanius, 2022). Awareness helps clients to determine whether their previous experiences are coloring their perception in the present moment and to distinguish between past and present. For those flooded by sensation and emotion, building tolerance of somatic sensation using top-down strategies is essential. For those wired to “shut down” or avoid somatic sensation as a defense mechanism, identification of somatic cues related to shifts in arousal and/or emotion may be facilitated through titrated somatic sensory feedback (Kearney and Lanius, 2022).

Being able to switch effectively between states can be seen as a sign of mental resilience: the ability to adapt and take control in the face of physical, emotional, and social challenges (Huber et al., 2016). Resilience refers to the ability to overcome stressful situations or recover from trauma. It also involves the ability to adapt to life changes through emotion regulation and to shape life in a meaningful way. Psychological problems often arise when a person is no longer able to adapt to these challenges. Related to this, flexibility means being able to regulate oneself after activation of the fight system and to reactivate the vagus nerve, so that one can calm down and recover. When the brain is under pressure, it works less efficiently; higher levels of cortisol hinder the functioning of the prefrontal cortex. This results in what is known as “tunnel vision”: a loss of perspective and impaired capacity for problem-solving, creativity, organization, and planning. Long-term thinking, risk assessment, and efficiency are all impeded by excess stress. Autonomic state biases reactions (neuroception) along a continuum of risk (Porges, 2023). Based on adversity history, reactions of the ANS may be biased toward maintaining a physiological state that supports defensive strategies (Kolacz et al., 2020).

For clients, to understand how these systems work requires psychoeducation: the therapist explains the autonomic states and how to recognize them in a compassionate and humane way.

### Polyvagal theory as a foundation for creative arts and psychomotor therapies

PVT could serve as an explanatory model for the underlying mechanisms of creative arts and psychomotor therapies. After all, PVT assumes a strong connection between body and mind, emphasizing neuroception and perception, self-regulation and co-regulation; the role of (un) conscious bodily signals and sensory experiences; and the need for safe social connection. PVT introduced the notion of somatic sensory processing, which encompasses vestibular and somatosensory processing and relates to the sensory systems concerned with how the physical body exists in and relates to physical space, as a major contributor to overall regulatory, social–emotional, and self-referential functioning (Kearney and Lanius, 2022). Creative arts and psychomotor therapies rely on the same mechanisms and emphasize working with and experiencing specific therapeutic tools (e.g., Malchiodi, 2012; Haeyen, 2018; Haeyen and Hinz, 2020; Malchiodi, 2020).

The bottom-up approach starts with sensing bodily signals, which creates space for emotional and cognitive meaning-making (Damasio, 2003; Gussak and Rosal, 2016; Haeyen, 2020; Malchiodi, 2020; Pénzes, 2023). Creative arts and psychomotor therapies actively seek to tap into these sensations, emotions, and other physiological signals, which are both informative and regulatory. By doing so, they have been found to reduce stress and cortisol levels (Solé et al., 2014; Kaimal et al., 2016). They enhance clients’ body awareness—which plays an important role in emotional experience and self-awareness (Damasio, 2010)—and sense of agency through movement, expression, and sensory experiences. Moreover, they can help clients to integrate cognition and emotion through creative and expressive processes, increasing their understanding of the interplay between thoughts, feelings, and behaviors (Damasio, 1994). Starting with cognition is not always the best place to start when restoring the self, post-trauma. In many cases, imagination and a sense of playfulness has been lost or diminished due to distress, anxiety, or dissociation (Malchiodi, 2020). Malchiodi described several reasons for including arts in trauma intervention: (1) letting the senses tell the story; (2) self-soothing mind and body; (3) engaging the body; (4) enhancing nonverbal communication; (5) recovering self-efficacy; (6) rescripting the trauma story; (7) making meaning; and (8) restoring aliveness.

In creative arts and psychomotor therapies, the “felt” sense, experienced within and through the body, is expressed in non-verbal, sensory-based, action-oriented artforms that tap the implicit embodied experiences of trauma defying narrative or logical expression (Terr, 1990; Malchiodi, 2020). Each form of creative arts and psychomotor therapies incorporates the physical body and meaningful, purposeful action which produces an intentional result, forming or re-forming sensory-motor feedback loops which engender a sense of agency, power, and positive self-environment relationship. The non-verbal rhythmicity inherent in music therapy may provide vibratory stimulation and an external source of rhythm when internal rhythms and sensorimotor synchrony is disrupted due to somatic sensory disintegration (Kearney and Lanius, 2022).

## Clinical application of polyvagal theory in creative arts and psychomotor therapies

The key processes in PVT that can be applied in creative arts and psychomotor therapies to activate the ventral vagal state and regulate the stress system are (1) noticing and naming, and (2) learning to change.

*Noticing and naming*: the individual learns to identify where they are on the stress ladder. This involves recognizing, exploring, and acknowledging the autonomic state, with a focus on safety as a condition for recovery. To this end, they experience and connect with bodily signals before attempting to make any changes.

*Learning to change*: the individual learns to influence their own autonomic state by paying attention to bodily experiences, sensations, and triggers, and learning to calm themselves, activate themselves, and make positive connections with others.

Connecting with these processes requires active attention from the individual concerned (e.g., Kalisvaart, 2022). According to PVT, an active vagus nerve is required to deploy the stress systems in a flexible way, thus increasing resilience. Creative arts and psychomotor therapeutic techniques could help to activate the vagus nerve so as to recognize different states and promote or prolong the desired state.

*Case vignette: psychomotor therapy/movement therapy (Jerry, age 34)*

*Jerry joined a psychomotor therapy group to improve his self-regulation and emotional wellbeing. The therapist started each session by guiding the group members through awareness exercises to help them connect with bodily sensations. Grounding techniques, such as mindful breathing or feeling the support of the ground, are used to activate the ventral vagus nerve and establish a sense of safety. In one session, the therapist invited the group members to work with a partner, performing mirrored or synchronized movements. These activities encourage social engagement and connection, also activating the ventral vagus nerve. Different group members were invited to take turns leading and following, fostering a sense of social safety. Gradually, the pairs themselves introduced more complex movements, encouraged by the therapist. The focus was on slowly expanding the individual’s comfort zone. Jerry and his partner pushed each other more and more firmly, while continuing to stay in balance. Given his fear of losing control of his impulses, he found this exciting. The therapist monitored Jerry’s as well as the others’ reactions, ensuring that the activities did not trigger a freeze, fight, or flight response. These exercises helped Jerry to notice both his own and his partners’ physical reactions, and enhanced his self-regulatory abilities and resilience.*

### Techniques for “noticing and naming”

*Awareness of physical cues* by practicing standing still and perceiving. A person may not be aware of, or accustomed to, a particular state of being, especially when under stress. Fostering relaxation involves learning to recognize the bodily signals of relaxation and cherishing those moments. To that end, the therapist draws attention to sensation, especially when something seems to change in the client: “Where do you feel this in your body? How does it feel? Is it a sense of pressure, a contracting, a tingling …?” The therapists can name what they see, calm the person down, or induce them to experience a physical change. Stress levels may be measured via heart rate and heart-rate variability. The link between vagus nerve activity and the high-frequency component of heart rate variability (HRV) correlating with vagal tone has been well established by Czamanski-Cohen et al. (2020). Paying attention to positive moments is important because our nervous system is particularly alert to insecurity, meaning positive signals are more likely to be neglected or ignored.

*Focusing or meditating* to connect with the individual’s physical and mental state and personal meaning. To this end, a range of mindfulness/attention exercises and working methods focus on stillness and cooperation between the ventral vagal and dorsal vagal state.

**Figure 4:**
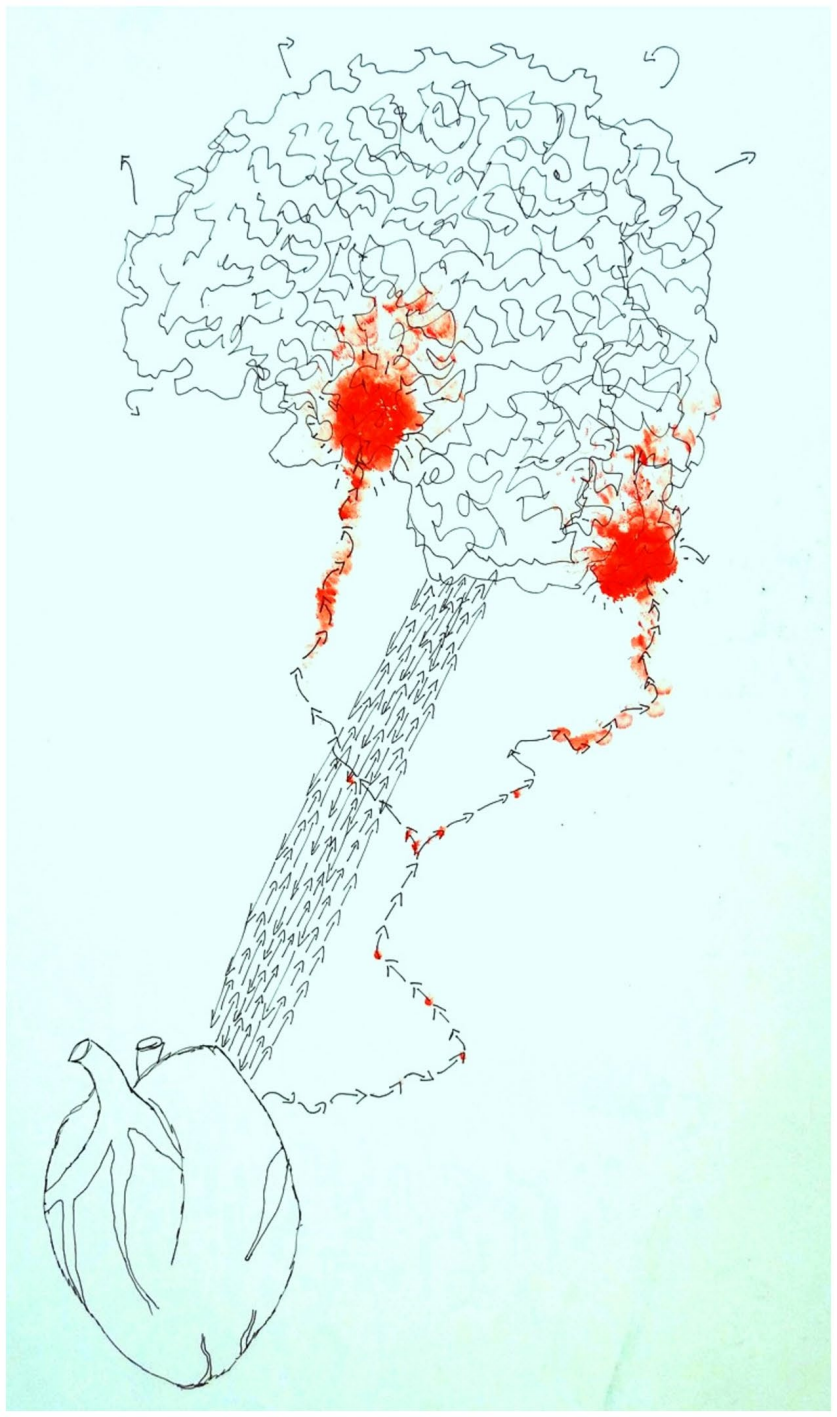
The two-way pathway between brain and heart in art therapy (John, age 43).

Figure 4 is an image showing the conceptual understanding of the connections between the heart and brain by a 43-year old male client in art therapy.

### Techniques for “learning to change”

#### Breathing techniques

Inhalation activates, exhalation relaxes. Breathing techniques focused on rhythm, tempo, and style of breathing (high in the chest, low in the belly) can influence the heart rate, increasing the ability to switch between activation and calmness. Creative arts and psychomotor techniques can be used to slow and deepen breathing; for example, drawing calm horizontal strokes with flowing watercolors, kneading clay more slowly, or playing the piano increasingly softly. The client and therapist can then explore what signals, emotions, and needs arise.

*Case vignette: art therapy (Ian, age 54) Ian had difficulty experiencing his emotions. He was quick to say, “I don’t know” and distance himself. He was asked to work with clay, with his eyes closed; an art-therapeutic method aimed at tactile sensory experience. The resulting uncertainty and dependence triggered a range of complicated signals and emotions regarding Ian’s relationship with his mother. He often felt angry towards her; he wanted to be emotionally independent but did not feel able. These feelings were now reflected in his relationship with the therapist. He became agitated: “Please tell me how to do this!” The therapist encouraged him to make contact with the material, to really feel the clay and observe what sensations and emotions arise. By slowing down, he was able to discern back pain, sadness, and anger. The therapist co-regulated this process, helping Ian to calm down and examine what the clay technique had triggered in him. In the subsequent verbal reflection, he connected it to the sense of threat he had often experienced in the relationship with his parents. That he had now dared to pay close attention to his senses and experiences enhanced his sense of autonomy and self-confidence.*

#### Mobilization

When someone feels “stuck” or unable to take action, the first goal is often to literally get them moving. This can be done through yoga, dance, or walking, but also by doing, creating something, or helping them feel heard. In the process of reparation from psychological trauma, various forms of bilateral stimulation or movement seem to be effective in engaging cross-hemisphere activity in the brain (Shapiro, 2001) and in art therapy possibly because it reconnects “thinking” and “feeling” (Malchiodi, 2012) via the sensory-based processes involved in art-making. A gradual build-up is important to maintain a sense of safety; for example, by visualizing first, taking small steps every day, or alternating between relaxing and strenuous forms of exercise.

#### Sensory activation of a desired state

Sensory feedback may, via interneuronal connections, provide additional portals to regulate the ventral vagus and functionally may act as a vagal nerve stimulator (Porges, 2023). This involves learning to dwell on sensory signals and use them in a focused way; for example, by focusing on music that evokes feelings of connection and safety (ventral vagal state) or activation (sympathetic system). It can include receptive techniques (listening to music/looking at art), engaging in play together, or creating a playlist for certain moments. The understanding of the adaptive function of middle ear muscles links listening to calming. It also provided the neurophysiological basis of an acoustic intervention known as the Safe and Sound Protocol (Heilman et al., n.d.). This protocol stimulates the ventral vagal complex to calm autonomic state, improve auditory processing, and stimulate spontaneous social behavior. The music of the SSP has been filtered through a patented, evidence-based algorithm that highlights specific sound frequencies that help regulate the autonomic nervous system and stimulate the vagus nerve. Music interventions have an overall significant effect on stress reduction in both physiological and psychological outcomes (De Witte et al., 2020). White noise or binaural beats at the desired frequency can be used to reduce stress or anxiety. Looking and feeling (touch) can also be relaxing or activating. The human body has approximately 11 million sensory receptors, about 10 million of which are dedicated to vision (Clear, 2018). Compared to any other sense, visual cues are thus the biggest catalyst of our behavior (Clear, 2018). Techniques need to be attuned to the individual: looking at art or working with a particular tactile material can have either a relaxing or activating effect depending on the person. This is especially so when deployed with a delay; for example, by painting ever more leisurely strokes on a generously sized page, kneading clay slowly, or working with a technique such as pottery. Similarly, people do not react in the same way to music or sounds. Perception is personal and requires tuning.

#### Relaxation and play

This requires careful attention to physical cues. Going into nature or using nature imagery/sounds can contribute to the sensory experience of relaxation. If a client has difficulty recognizing or expressing emotions, the therapist can offer techniques to promote the experience of relaxation, playfulness, and other positive emotions. Experiencing rather than avoiding these emotions creates space for the client to experience negative emotions as well. Techniques that combine a ventral vagal and sympathetic state can foster play. In group settings, the social engagement system is used to regulate mobilization.

#### Positive experiences

The ANS is particularly alert to insecurity, meaning positive signals are more likely to be neglected or ignored. In art therapy, a positive moment from the present or past can be played out, imagined, or vocalized. Clients can keep a (visual) diary to retain and reinforce positive experiences: they can record experiences they found nice or touching, things they are grateful for, or things they might do for someone else. Techniques, materials, and resources can be offered so as to promote relaxation, enjoyment of play, and other positive emotions. Daring to let positive emotions in teaches clients that they need not avoid emotions, and creates space to experience and redirect negative emotions as well.

*Expression of emotion*s through making music, singing, dancing, playing, visual art, etc. Singing, for example, is a powerful neural exercise involving both middle ear muscles, mouth muscles, pharynx, face, and trunk. It requires longer exhalations, thus regulating the breathing and having a positive effect on the vagus nerve. Singing, painting, and engaging in joint play activates the social engagement system and creates connection with others. Likewise, seeing, hearing, and challenging one another fosters co-regulation, leading to connection.

#### Insight into where signals come from and how they influence our actions

This requires exploring which stress system and underlying pattern is triggered when, and where it originated in the past. Understanding and giving meaning to such systems and patterns helps to strengthen the client’s identity and self-awareness. Techniques that shape actions in the present include imagining and depicting a positive moment from the past or keeping a positive (visual) diary. The aim is to deliberately evoke and reinforce a positive affect.

### The therapeutic relationship

The therapeutic relation is very important for activation of the ventral vagus branch. Underlying every interaction there is an inherent urge to connect, to engage in an experience of interpersonal contact. This most basic urge to connect can be divided in three responses; orienting toward or away from interpersonal contact, an affective response to the contact, and an associated, affectively-loaded, seeking drive. These responses depend on specific brain circuits and dopamine pathways (Corrigan and Christie-Sands, 2020). The helpful therapeutic relationship may be characterized as careful, mindful, with attention to orienting-tension-affect-seeking sequences when the therapist and the client collaborate on eliciting and describing them. When the therapeutic relationship is based on safety and support, clients can express themselves freely in order to increase social engagement. In terms of co-regulation of the client, it is important for the therapist to be aware of where they themselves are on the stress ladder. After all, nervous systems resonate with each other. Knowledge of how breathing, intonation, and prosody send signals of safety is essential (Dana, 2020). In attentive listening, for example, tilting the head toward the client affects how they feel heard. The therapeutic relationship is important in relation to activating the ventral vagus branch. It can include the therapist and client, as well as clients in a therapy group.

*Case vignette: music therapy (Cecilia, age 27) Cecilia entered the room looking restless. Her body language and facial expression suggested an agitated physiological and emotional state. When the music therapist asked how she felt, she said she was angry. The therapist invited Cecilia to begin a rhythm activity using simple percussion instruments and a predictable rhythm, which provided a stable, structured experience. The therapist performed the same rhythm in synchrony, which stimulated the ventral vagus and promoted a sense of safety through co-regulation. Then the therapist invited Cecilia to sing with her, activating the system of social engagement. Soon a kind of battle song emerged, with melodic and rhythmic patterns that matched her current state. Gradually they shifted to more regulatory and calming patterns. This method promoted self-expression and emotional release. After this, the therapist asked Cecilia what instrument she would like to play next, giving her a sense of control and empowerment. For Cecilia, autonomy meant safety and social connectedness. Together they played the piano. After a while, Cecilia began to play a sad melody, accompanied by the therapist. Finally, they reflected on the process. Cecilia verbalized and explored her emotional experiences during the session, reinforcing the integration of the therapeutic process. She connected with an emotional layer beneath the anger and explored with the therapist what had made her feel sad that day.*

## Discussion

PVT can help creative arts and psychomotor therapists to better understand the neurobiological processes underlying creative expression, active doing, and emotional wellbeing. It may help them to interpret personal and therapeutic processes and to design effective, appropriate interventions. Conversely, creative arts and psychomotor therapies offer opportunities to apply the theory in practice in a manner suited to the sensory, active, and body-oriented perspective of PVT. The strength of these therapies lies in their predominantly bottom-up approach: becoming aware of the body’s signals creates space for new emotional and cognitive meaning. With its focus on the connection between body and mind, conscious and unconscious bodily signals, sensory experiences, and the need for safe social connection, PVT can hypothetically explain the working mechanisms underlying creative arts and psychomotor therapies.

Anecdotal evidence suggests that PVT and the creative arts and psychomotor therapies form a promising combination. Bonilla (2020) reports that it helped people with a history of abuse feel more in touch with their bodies and understand how and when they were triggered. Others have applied PVT in dance and movement therapy (Wagner, 2015; Gray, 2017) and yoga (Sullivan et al., 2018). Magsamen and Ross (2023) indicate that sensory stimulation during art making, which is not chaotic, but which feels safe and new—even for just 20 min a day—can help to create new nerve connections in the brain.

Other psychological theories connect to the same building blocks as PVT. Compassion-focused therapy (CFT; Gilbert, 2009, 2014), for example, relies on the same evolutionary stress systems, helping people to reduce their own stress levels and experience (self-) connection, warmth, safety, and reassurance by developing a supportive inner voice. Compassion focused therapy is rooted in an evolutionary, functional analysis of basic social motivational systems (e.g., to live in groups, form hierarchies and ranks, seek out sexual, partners help and share with alliances, and care for kin) and different functional emotional systems (e.g., to respond to threats, seek out resources, and for states of contentment/safeness). In addition, about 2 million years ago, (pre-)humans began to evolve a range of cognitive competencies for reasoning, reflection, anticipating, imagining, mentalizing, and creating a socially contextualized sense of self. These new competencies can cause major difficulties in the organization of (older) motivation and emotional systems. CFT suggests that our evolved brain is therefore potentially problematic because of its basic ‘design,’ being easily triggered into destructive behaviors and mental health problems (called ‘tricky brain’). However, mammals and especially humans have also evolved motives and emotions for affiliative, caring and altruistic behavior that can organize our brain in such a way as to significantly offset our destructive potentials. CFT therefore highlights the importance of developing people’s capacity to (mindfully) access, tolerate, and direct affiliative motives and emotions, for themselves and others, and cultivate inner compassion as a way for organizing our human ‘tricky brain’ in prosocial and mentally healthy ways (Gilbert, 2014). Compassion focused art therapy is developed for people diagnosed with a cluster B/C personality disorder as they often lack self-compassion skills (Haeyen and Heijman, 2020).

Various scientific theories focus on how our brain, nervous system, and other organs are related to our psychological functioning under stress. Some critiques of the popular PVT point out that the basic assumptions of the theory are untenable (Steffen et al., 2022; Grossman, 2023; Verkuil, 2023). Steffen and colleagues propose a new evolutionarily based model, the adaptive brain, that is founded on adaptive prediction resulting from interdependent brain networks using interoception and exteroception to balance current needs, and the interconnections among homeostasis, allostasis, emotion, cognition, and strong social bonds in accomplishing adaptive goals (Steffen et al., 2022). The evolutionary, neuroanatomical, and neurophysiological aspects of PVT have been misunderstood or misinterpreted, with researchers challenging the distinction between reptiles (with only a dorsal vagus) and mammals (polyvagal, with an evolved, “new,” and “smart” ventral vagal social-connection system) (Neuhuber and Berthoud, 2022; Taylor et al., 2022). They posit that the “new” vagus is neither particularly novel nor unique to mammals; indeed, many animals are “polyvagal.” They also contest the distinction between dorsal and ventral vagal responses and the claim that the freezing response, including the drop in heart rate, is determined by the dorsal vagal motor nucleus in the brain stem. Stimulating this nucleus has little effect on the heart rhythm, which is determined by many different cranial nerves and areas. Thus, critics assert that the role of the vagal nerve is overestimated and the system more complex than suggested by PVT (Neuhuber and Berthoud, 2022; Taylor et al., 2022). Porges (2021) himself critically reflects on the PVT and also provides the scientific foundation for the testing of hypotheses generated by PVT in a recent publication (Porges, 2023). These offer an optimistic possibility of a more informed level of scientific discourse that would further explore the important relationships between the ANS and human experience that have been highlighted by PVT.

In conclusion, PVT provides an understanding of the core features of the mammalian ANS needed to co-regulate and trust others. It also provides insights into the consequences of autonomic state for mental and physical health. The vagal nerve can still serve as an important conduit of social communication, if not the driver. The psychological aspects that PVT associates with the vagus (such as safety, connectedness, and the role of the body in the face of stress and threat) are certainly relevant in therapeutic settings. At the least, PVT provides a metaphor to frame and understand what is happening in the stress system and improve one’s capacity to deal with it. Experiences of clients and therapists in clinical practice show that the model offers a useful, easy-to-understand framework. PVT gives a voice to the personal experiences of individuals who have experienced chronic threat (i.e., trauma and abuse) or illness and structures an optimistic journey toward more optimal mental and physical health. It is this core, described by PVT, that links our biological imperative to connect with others to neural pathways, via neuroception, that calm our ANS (Porges, 2023).

Should treatment be focused solely on established evidence-based theories and treatment guidelines? Such an approach would stymie innovation. What is more, the explanatory model underlying recommended treatments is often notably weak. If PVT-based interventions could increase treatment effectiveness and rate, they warrant further investigation.

What is clear is that psychological pain is accompanied by physical reactions, and paying attention to clients’ physical cues and degree of arousal contributes to recovery. PVT offers an appealing, if not comprehensive, explanatory, hypothetical model for the contribution of the creative arts and psychomotor therapies to regulating stress and emotion and dealing with trauma, where restoring regulation of the ANS is key. The creative arts and psychomotor therapies, conceptualized in line with PVT, facilitate mind–body interventions as tools for restoration of an embodied self, strengthening resilience in a way that is understandable and applicable to those seeking personal recovery.

## Data availability statement

The original contributions presented in the study are included in the article/supplementary material, further inquiries can be directed to the corresponding author.

## Ethics statement

Written informed consent was obtained from the individual(s) for the publication of any potentially identifiable images or data included in this article.

## Author contributions

SH: Writing – original draft, Writing – review & editing.
